# Restriction of the family-centered care miracle by COVID-19: the role of the father in sleep disturbance of hospitalized pediatrics

**DOI:** 10.5935/1984-0063.20220071

**Published:** 2022

**Authors:** Sajjad Khaksar, Faramarz Kalhor

**Affiliations:** Faculty of Nursing and Midwifery, Isfahan University of Medical Sciences - Isfahan - Iran

**Keywords:** Fathers, Pediatrics, Pneumonia, Sleep, Family-Centered Care

## Abstract

**Introduction:**

One of the important outcomes of hospitalization in infants with respiratory disease is sleep disturbance. This study was performed to investigate the role and participation of fathers in the sleep of hospitalized pediatrics with pneumonia during the COVID-19 pandemic.

**Material and Methods:**

In this clinical trial study, the parents of 40 children aged from 12 to 36 months hospitalized with a diagnosis of pneumonia were randomly assigned to the control and intervention groups. After implementing a supportive training program for the fathers in the intervention group, each father made an online video communication with the infant and the mother as the primary caregiver. Mothers completed the brief infant sleep questionnaire-revised at the beginning and a week later.

**Results:**

Data analysis showed that daytime sleep duration, setting time and total 24-hour sleep time in the intervention group improved compared to the control group. In addition, parents’ behavior toward their child’s sleep improved in the intervention group compared to the control group, but parents’ perceptions of their child’s sleep in the intervention group did not show a significant difference compared to the control group. The mean total score of the intervention group also improved compared to the control group.

**Conclusion:**

Planning to maintain family unity by educating and supporting fathers during the child’s illness and their paternal support during the COVID-19 pandemic can improve the sleep of the hospitalized child due to pneumonia.

## INTRODUCTION

Adequate sleep plays an important role in the optimal growth and development of children^1^. Studies have shown that there is an association between adequate sleep and physical growth and memory, overweight, obesity, language, and executive functions^1,2^. In this regard, organizations such as WHO and the National Sleep Foundation (NSF) have published guidelines and recommendations for children’s sleep^3,4^. In sick children, adequate sleep can be more important because adequate sleep promotes protein synthesis, facilitates cell division and tissue repair mechanisms, and can improve the healing process in sick children^5^.

Despite the many benefits of adequate sleep for sick children, but sleep disturbance has unfortunately been cited as one of the important outcomes of hospitalization in children and infants with respiratory diseases^6,7^. Pain, discomfort, medications, stress and anxiety, environment noise, change of environment, separation from other family members, loud noises, warnings and frequent visits of nurses according to the hospital routine, medical staff talking to other patients, lighting lights and cough in children with respiratory disease (REM sleep disorder) have been reported as the causes of sleep disorders in hospitalized children^7-9^. A study reported the rate of sleep disorders in hospitalized infants is about 30%^10^. However, waking up overnight in toddlers reduces total sleep time during the second year^11,12^.

Nocturnal awakening is usually less frequent in toddlers than infants, but most children in this age group have at least one weekly wake-up in which they inform their parents of the awakening^13^. When children have difficulty falling asleep, their parents also have difficulty sleeping^14-16^. Hospitalized children both physically and emotionally need the simultaneous presence of their parents when they are hospitalized, and in the presence of both of them, the child feels more relaxed and the parents play an important role in the child’s life during hospitalization^17^.

Many studies have reported that the majority of participants in parental interventions are mothers and even do not provide information on the extent of the father’s involvement^18,19^. In one study, fathers’ participation in childcare and hospitalization programs was reported to be about 13-21%^20^. However, according to the concept of family-centered care, parents should be involved in all therapeutic care for the sick child inside and outside the hospital^21,22^.

Although mothers and fathers influence their children in similar ways by considering competence in social interactions, scientific progress, and mental health, however, fathers’ participation has a different role and nature compared to mothers^22^. So that there is a significant relationship between children’s growth and development and fathers’ participation in their children’s sleep and their relationship with their child. Several studies have reported the effect of paternal factors on children’s sleep^23,24^. A study reported a decrease in nocturnal awakening with greater father involvement in 6-month-old children^24^. Another study reported that mothers saw more sleep problems in their children in the absence of fathers^25^. However, with the hospitalization of the children and the absence of the father, the children felt fear and loneliness and requested the presence of the father^26^. Children whose parents were present in the hospital at the same time tended to spend less time alone awake, crying, and had more social interactions with family members than with staff^27^.

This absence of fathers becomes more important when the results show that after infants were admitted to the ICU, mothers (93%) reported more severe sleep disorders and fatigue than fathers (60%). Actigraph reports also indicated that mothers experienced much more waking hours during the night than fathers^28^. All of this may prevent the mother from adapting to the hospital environment and supporting her child, which can have a negative effect on the child^29^. However, poor parental bonding is also associated with higher levels of parental stress^30^. Not only does it negatively affect the child’s outcomes, but it also affects the parents themselves. In this regard, family-centered interventions and involvement of fathers in the process of caring for infants can have significant effects on improving the support of mothers and subsequently, the child^31^.

However, the absence of the father and its outcomes on the child’s health has not been widely studied and fathers are a missing link in family-centered care^32^. On the other hand, given the widespread prevalence of COVID-19, physical or social distancing is the best way to reduce disease transmission; therefore, to ensure the safety of patients, staff and family members, some pediatrics hospitals have restricted the presence of family members. In this situation, ambiguity is created for committed staff on how to adhere to the principles of family-centered care^33,34^. Therefore, rapid adaptation of family-centered methods and tools is essential to circumvent the limitations of physical presence. One of the best ways is to use video communication technology^35^. Therefore, the researcher conducted the present study to evaluate the effect of the role and participation of fathers on the sleep of hospitalized children with pneumonia during the COVID-19 pandemic.

## MATERIAL AND METHODS

The study protocol was approved by the ethics committee of Isfahan University of Medical Sciences (IR.MUI.RESEARCH.REC1399.1360). To comply with the ethical standards, after providing sufficient information to the units participating in the research, written informed consent was obtained from the child’s parent or legal guardian. Privacy and confidentiality of information were considered throughout the research process.

### Study groups

This study is a clinical trial that was registered in Iran Clinical Trial Center (https://www.irct.ir) with the number IRCT20210421051029N1. The study included intervention, control, pre-test and post-test groups and was performed in the selected hospitals of Isfahan University of Medical Sciences. The study samples consisted of 44 children aged 12 to 36 months with a diagnosis of pneumonia hospitalized in the pediatric wards and their parents who were randomly assigned to the control and intervention groups using the randomized allocation software (RAS) program with the same blocks (n=22 in each group). Two sample from each group were excluded from the study because did not cooperate to perform the intervention and to complete the questionnaire.

Inclusion criteria were: child’s age of 12 to 36 months, the first day of hospitalization of the child with a diagnosis of pneumonia, not receiving specific medication for insomnia problems, parents’ ability to use video calls and the presence of the father in the family (did not die). Exclusion criteria included absence and nonparticipation of the father in two or more sessions, dissatisfaction and the unwillingness of the child’s family to continue the study, increasing the severity of the disease so that the child is unable to participate in the study, take sleeping pills or drugs with the hypnotic potential death of the child, discharge of the child before the end of the supportive training program and change of diagnosis in the child.

To calculate the sample size, we used the relevant formula that the minimum sample size of 20 people in each group was obtained with a 95% confidence interval and 80% test power coefficient, that considering to 10% loss, 22 people were considered in each group.

### Procedure

For the fathers in the intervention group, intervention measures were performed in two stages, the training stage and the supportive stage. The supportive training program included educating and supporting fathers who had a hospitalized child with pneumonia, and the mother was the infant’s primary caregiver. For this purpose, a 90-minute face-to-face individual training session was held that includes information about the child at infancy (behaviors and physical symptoms) and the child’s diseases, the effect of hospital conditions on the child, teaching the role of the father in supporting and caring for the child physically and emotionally, also tips on how to interact with children through video calling.

After this 90-minute meeting, an internet package was provided for the parents of the intervention group, and to support the fathers, exchange information between parents and researchers was performed with the participation of parents in social networks (WhatsApp) virtually for 4 consecutive days (twice a day) and with prior coordination with the researcher.

To support fathers, fathers spoke virtually or in person about their experiences and feelings about their child being hospitalized and about the problems their child experienced in being hospitalized. There was also time to answer the questions. Before video communication with the child, the educational content includes information about normal sleep patterns, children’s sleep needs, symptoms and outcomes of sleep disorders, sleep hygiene information, and a predicted guide about the child’s sleep during hospitalization was virtually educated to the fathers. So, the fathers performed the training for their child through video calls with the cooperation and supervision of the researcher, twice a day (once before bedtime) for 15 to 30 minutes each time^36^.

Fathers used relaxing behaviors and skills to prevent or reduce sleep problems. In addition, fathers were asked to bring with them material as well as storybooks containing colorful pictures and fun characters, musical instruments, their child’s latest artwork such as a painting, or whatever they intended to show him. Not only does it keep the child interested in communicating with the parents, but it also helps to calm the children. At the end of the video call, people could play music and sing with each other or play age-appropriate games, and at the end, they were asked to use a “kiss” to say goodbye. The control group also received routine care.

### Data collection

The questionnaires were completed by the mother on the first day of the child’s hospitalization and 7 days after completing the first questionnaire. Data were collected using a demographic questionnaire and the brief infant sleep questionnaire-revised (BISQ-R), which assessed sleep patterns, parental perception, and sleep-related behaviors in infants up to 36 months of age in the past week.

Infant sleep (IS) score includes 5 questions about sleep patterns, including sleep onset delay, number and duration of waking up at night, longest sleep duration and total nighttime sleep. Parental perception (PP) score includes three questions about caregiver perception of sleep problems, nighttime sleep, and child’s general sleep problems. The subscale of parent behavior (PB) score includes 11 questions about sleep ecology, including daily sleep consistency, sleep, parental behavior at the time of sleep onset and after waking up at night, and the sleeping place at the time of sleep onset and after waking up at night. Higher scores indicate parental behaviors, which reinforce healthy sleep behaviors and independent sleep in the infant. The total BISQ-R score is also calculated from the mean of three subscales.

The face and content validity of this tool as assessed by experts and also re-test r=0.848 (*p*<0.001) and kappa coefficient 0.939 were used to calculate the reliability; also (95%CI: 0.858-1.00, (*p*<0.001) were used for agreement between self-management and clinical interview^37,38^.

Strong correlations were reported between repetition of sleep assessment for nighttime sleep duration (r=0.82), daily sleep duration (r=0.89), number of night awakenings (r=0.88), night awakening duration (r=0.95), time of night sleep onset (r=0.95) and sleep time (r=0.94). BISQ-R criteria are significantly related to sleep criteria obtained from actigraphs and daily sleep reports. High correlation of retest (r>0.82) was shown for BISQ-R criteria for a subsample of 26 infants^39^. Cronbach’s alpha was calculated to be 0.853 in this study. External reliability (test-retest), Pearson correlation coefficient in three subsets of child sleep was r=0.6, parental perception r=0.866, parental behavior r=0.5, and in total r=0.76.

### Statistical analyses

Data were analyzed using SPSS software version 18 at alpha level of 0.05. Paired t-test, independent t-test, Fisher’s exact test and analysis of variance were used to calculate the differences between the control and intervention groups before and after the intervention. It should also be noted that four samples that did not complete the initial questionnaire were excluded from the study.

## RESULTS

The mean age of the children participating in this study was 24.5±3 months; 65% were boys and 35% were girls. The mean age of mothers was 29.7 years. According to the chi-square test, the two groups were not significantly different in terms of child’s age, mother’s age and child’s gender (*p*>0.05) (Table 1).

**Table 1:** Demographic characteristics in the intervention and control groups at baseline [Mean (SD), Freq. Number (%) of participates, n=40].

		Intervention	control	P
Child age	**Month**			0.452^*^
	12-18	4(20%)	6(30%)	
	18-24	6(30%)	5(25%)	
	24-30	3(15%)	3(15%)	
	30-36	7(35%)	6(30%)	
	Mean (SD)	24.9(3.02)	23.95(3)	
Sex	Male	14(70%)	12(60%)	0.405^**^
	Female	6(30%)	8(40%)	
Mother age	**Years**			0.956^*^
	20-24	2(10%)	4(20%)	
	25-29	6(30%)	8(40%)	
	30-34	9(45%)	7(35%)	
	35-39	2(10%)	0(0%)	
	40-44	1(5%)	1(5%)	
	Mean (SD)	30.55(3.45)	27.6(3.39)	

Notes:

* Independent t-test;

** Chi-square.

### Child sleep

The results showed that after the intervention, improvement occurred in daytime sleep and total sleep time in the intervention group compared to the control group (*p*<0.05). The results showed no significant difference between the intervention and the control groups in terms of nocturnal sleep time, night awaking, number of night waking, longest continuous sleep period, and setting time (Table 2).

**Table 2:** Sleep-wake patterns for infants and toddlers.

			Mean(SD)	F	p^*^	ES^**^
Day time sleep (hour)	Control	Before	2.6(1.14)	5.654	0.001	0.182
		After	2.8(1.1)			
	Intervention	Before	2.65(1.04)			
		After	3.8(.95)			
Nocturnal sleep time (hour)	Control	Before	10.02(.69)	0.688	0.562	0.026
		After	9.67(.65)			
	Intervention	Before	9.82(1.24)			
		After	9.97(.65)			
Bedtime routine	Control	Before	10.02(0.69)	0.688	0.562	0.026
		After	9.67(0.65)			
	Intervention	Before	9.83(1.24)			
		After	9.97(0.65)			
Sleep onset latency (min)	Control	Before	20.5 (8.41)	2.252	0.089	0.082
		After	16.25 (4.55)			
	Intervention	Before	20.5 (8.72)			
		After	16 (7.71)			
Duration of night waking (min)	Control	Before	31.25(22.3)	1.047	0.377	0.040
		After	23(16.65)			
	Intervention	Before	24.75(11.4)			
		After	24(12.73)			
Total sleep time (hour)	Control	Before	12.63(1.56)	3.619	0.017	0.125
		After	12.48(1.23)			
	Intervention	Before	12.48(1.93)			
		After	13.78(1.03)			
Number of night waking	Control	Before	2.85(0.74)	2.358	0.78	0.085
		After	2.75(0.85)			
	Intervention	Before	2.65(0.8)			
		After	3.3(0.93)			
Longest continuous sleep period (hour)	Control	Before	8.02(0.69)	0.688	0.562	0.026
		After	7.67(0.65)			
	Intervention	Before	7.82(1.25)			
		After	7.97(0.66)			
Number of nap	Control	Before	1.35(0.67)	2.704	0.051	0.096
		After	1.3(0.47)			
	Intervention	Before	1.75(0.63)			
		After	1.6(0.5)			

Notes:

* One-way ANOVA;

** a. Eta-squared and Epsilon-squared are estimated based on the fixed-effect model;

** b. Negative but less biased estimates are retained, not rounded to zero.

### Bedtime and falling asleep (setting time)

There was no significant relationship in bedtime routine in the intervention group after the intervention. Also, there was no significant difference in bedtime routine between the two groups after the intervention and the children in the intervention group did not have an earlier bedtime routine compared to before the intervention and the control group (F=0.688, ES=0.026, *p*=0.562). There was no significant difference in sleep onset latency between the two groups and a decrease in sleep onset latency was observed in both groups before the intervention compared to after the intervention (F=2.252, ES=0.082, *p*=0.089).

### Night waking and sleep consolidation

No statistically significant difference and improvement were observed in the intervention group compared to the control group in terms of duration of night waking time, number of night waking and longest continuous sleep period after the intervention. In the intervention group, the duration of night waking time was 24 minutes and in the control group was 23 minutes (F=1.047, ES=0.0403, *p*=0.377). The number of night waking after the intervention in the intervention group was average 3.3 times and in the control group was 2.75 times (F=2.358, ES=0.0853, *p*=0.78). Although the longest continuous sleep period after the intervention in the intervention group showed an increase of 18 minutes compared to the control group, but was not statistically significant (F=0.688, ES=0.026, *p*=0.562).

### Sleep duration

There was a significant time difference in improving day time sleep after the intervention in the intervention group compared to the control group (F=5.654, ES=0.182, *p*=0.001), so that the children in the intervention group had 60 minutes more daily sleep than the control group. However, although nocturnal sleep time in the intervention group after the intervention showed an increase of 18 minutes compared to the control group, but was not statistically significant (F=0.688, ES=0.026, *p*=0.562). Significant differences were also observed in the total sleep time after the intervention in the intervention group compared to the control group (F=3.619, ES=0.125, *p*=0.017), so that the children in the intervention group had 78 minutes more total sleep time than the control group. There was no significant difference in the number of naps in the intervention group after the intervention compared to the control group (F=2.704, ES=0.096, *p*=0.051).

In general, the mean score of infant sleep (IS) in the intervention group after the intervention differed significantly from the control group (*p*<0.05). The mean score of infant sleep (IS) was not statistically significant before and after the intervention in the intervention group (*p*>0.05) (Table 3).

**Table 3:** Infant sleep (IS), parent behavior (PB), and parent perceptions (PI) in three BISQ-revised subscales.

	Groups	Before	After	p^*^
Infant sleep	Intervention	52.77(10.88)	54.52(7.82)	0.025
	Control	56.89(10.44)	56.36(12.39)	0.018
		T=-1.221 ^**^p=0.230	T=-0.564 ^**^p=0.576	
Parent perception	Intervention	61.46(20.9)	69.88(17.33)	0.213
	Control	60.38(20.52)	57.38(18.78)	0.496
		T=0.165 ^**^p=0.869	T= 2.187 ^**^p=0.035	
Parent behavior	Intervention	53.94(7.24)	59.98(8.93)	0.493
	Control	57.18(7.57)	56.37(7.95)	0.633
		T=-1.384 ^**^p=0.174	T=1.350 ^**^p=0.185	
Overall	Intervention	56.05(7.57)	61.45(7.87)	0.849
	Control	58.15(8.57)	56.7(8.23)	0.850
		T=-0.818 ^**^p=0.418	T=1.866 ^**^p=0.070	

Notes:

* Paired t-test;

** Independent samples test.

### Caregiver behavior and perceptions

In terms of falling asleep, there was no difference between the infants in the intervention and control groups after the intervention (*p*>0.05). Also, mothers in the intervention group reported improved sleeping well of their children after the intervention (*p*<0.05). In statistical comparison after the intervention between the two groups, the statistical results showed a significant difference that indicated an improvement in sleeping well reported by the mothers of children in the intervention group compared to the children in the control group (*p*<0.05). Regarding feeding to sleep, mothers in the intervention group reported they used 5% less feeding to sleep for their children after the intervention than before the intervention. In comparison between the two groups after the intervention, although the mothers of the intervention group used less feeding to sleep for their children, but no statistically significant difference was observed (*p*>0.05).

There was a statistically significant difference after the intervention between the intervention group and the control group when children woke up at night and parental behavior (*p*<0.05), so that mothers used more breastfeeding for their infants to sleep. Also, the use of Bottle of milk was less in the mothers of the intervention group than in the control group. Also, they less pick up their children and put him on his back while he is still awake (Table 4).

**Table 4:** Parent behavior and perception for infants and toddlers sleep.

Grouping of sample		Control (Percent)		Intervention (Percent)		p^*^
		Before	After	Before	After	
When your child wakes up during the night, what do you usually do?	Pick up my baby and put him on his back while he is still awake	10.0%	55.0%	55.0%	20.0%	0.001
	Give a bottle of milk to put him to sleep	45.0%	40.05%	25.0%	20.0%	
	Breast milk	40.0%	5.0%	20.0%	45.0%	
	None of these	5.0%	0%	0%	15.0%	
Consider sleep a problem	Not a problem at all	50.0%	30.0%	50.0%	45.0%	0.016
	A very small problem	5.0%	20.0%	5.0%	25.0%	
	A small problem	30.0%	35.0%	30.0%	20.0%	
	A serious problem	15.0%	5.0%	15.0%	10.0%	
Falling asleep	So hard	45.0%	30.0%	50.0%	45.0%	0.218
	It is somewhat difficult	0%	20.0%	5.0%	25.0%	
	Neither easy nor difficult	50.0%	35.0%	30.0%	20.0%	
	Somewhat easy	0%	5.0%	0%	0%	
	Very easy	5.0%	10.0%	50.0%	10.0%	
Feeding to sleep	Yes	20.0%	15.0%	40.0%	35.0%	0.279
	No	80.0%	85.0%	60.0%	65.0%	
How well does your child usually sleep at night?	Very well	0%	0%	5.0%	5.0%	0.013
	Well	25.0%	30.0%	35.0%	55.0%	
	Fairly well	50.0%	45.0%	40.0%	30.0%	
	Poorly	20.0%	10.0%	15.0%	10.0%	
	Very poorly	5.0%	15.0%	5.0%	0.0%	

Notes:

* Fisher exact test.

In general, the mean score of paternal perception (PP) in the intervention group was not statistically significant before and after the intervention (*p*>0.05). In comparison of the mean score of paternal perception in the intervention group after the intervention, there was no statistically significant difference compared to the control group (*p*>0.05). Although there was a statistically significant difference in the mean score of paternal behavior (PB) towards children’s sleep in the intervention group before and after the intervention (*p*<0.05), but after the intervention, no significant difference was observed in the mean score of paternal behavior in the intervention group compared to the control group (*p*>0.05) (Table 4).

## DISCUSSION

Sleep disturbance has been mentioned as one of the important outcomes of hospitalization in children and infants with respiratory diseases^6,7^. Therefore, hospitalized children need the simultaneous presence of their parents, both physically and emotionally, when they are hospitalized. The child feels more relaxed in the presence of both mother and father^22^. The Father’s role as a member of the family plays an important. In the participation of fathers in the care of their hospitalized children, benefits have been reported for the child^20,39^.

Involvement and education of fathers are essential to adhering to family-centered care principles in situations where some pediatrics’ hospitals have restricted the presence of family members to reduce transmission of COVID-19^2,34^. Therefore, in an online intervention for fathers in the present study, the results showed that the educational-supportive intervention of fathers is beneficial in improving various aspects of sleep in hospitalized infants and toddlers with pneumonia, including daytime sleep, total sleep time, and mothers’ behavior toward children’s sleep. No significant improvement was observed in duration of night waking time, number of night waking and longest continuous sleep period, although the longest continuous sleep period increased by 18 minutes. Perhaps the reason for no significance is the hospital environment because the sleep-disturbing factors in the hospital, such as frequent visits of nurses, the presence of several patients’ beds in one room and other factors affect night waking and sleep consolidation. Also, mothers’ behavior toward their children’s sleep improved following the intervention.

Although there was no significant relationship between mothers’ perceptions of their children’s sleep, but mothers reported improvement in their children’s sleep, and mothers reported fewer sleep problems during their children sleeping; however, difficult sleeping was still reported in children. Given these cases, it seems that in future studies, in addition to increasing the duration of such interventions, more samples will be examined.

Restriction of the fathers’ presence due to the COVID-19 pandemic has led to the decrease in family care in hospitals. Therefore, although in some cases, no improvement was observed in some items, but online and virtual interventions in such conditions can be an important source in improving family-centered care and fathers’ participation in the hospital and affecting their children’s sleep. Also, the improvement of some sleep items in children in the control group was an interesting issue, which could indicate the effect of routine care on the sleep of hospitalized children, which may reflect the actions that parents take towards their children’s sleep.

In the present study, to perform the intervention, the same training was given to all fathers participating in the study and they were asked to use relaxing behaviors and skills in visual contact to prevent or reduce the occurrence of sleep problems. In addition, fathers were asked to have the storybooks which included colorful pictures and fun characters, musical instruments, their child’s latest artwork such as a painting, or whatever the child intended to show him. This not only makes the child continue to communicate with the parents, but it also leads to calmness in the children.

In this regard, the study by Leichman et al., in 2020^40^, examined the effect of a sleep-behavioral intervention provided to caregivers via smartphones, the results showed that the total 24-hour sleep score improved like as in the present study. In addition, infants’ caregivers had a better understanding of their children’s sleep, but in the present study, mothers’ perceptions of their child’s sleep did not change. Mindell et al., in 2011^41^, examined the impact of an Internet-based intervention on infant and toddler sleep disorders, which, like the present study, improved children’s sleep, including a significant reduction in sleep-disordered behaviors and a significant improvement in sleep onset delay, number and duration of night waking.

Papaconstantinou et al. (2018)^42^ examined the effect of a behavioral-educational intervention to enhance children’s sleep during hospitalization like as the present study, the results reported an improvement in the quality of sleep in hospitalized children. The study conducted by Santos et al. (2019)^43^, which investigated the effect of parental counseling on sleep habits of healthy children showed that unlike the present study, the educational intervention did not improve the sleep of infants, although the mean age of children in the present study was 24 months, but the study of Santos et al.^43^ was performed on 12-month children. Also, unlike the Santos’ study^43^, the present study was performed on hospitalized children. Also, in the two studies conducted by Spilsbury et al. (2004)^44^ and Liu et al. (2000)^45^ to investigate the cases that affect the sleep of primary school children, unlike the present study, no significant relationship was observed in the participation of fathers on the quality of children’s sleep; while in the present study, fathers were effective on the improved quality of sleep in their hospitalized children. The differences between the present study and the above two studies include the age group of the participating children and the research environment.

The above studies showed that despite the effect of supportive educational programs in improving children’s sleep, the role of fathers as a member of the family has been neglected in interventional programs for family-centered care of their children’s sleep. The present study, like the above studies, showed that supportive educational programs can be effective on children’s sleep, but unlike two studies conducted by Spilsbury et al. (2004)^44^ and Liu et al. (2000)^45^, showed that fathers’ participation affects their children’s sleep; fathers like mothers can improve their children’s sleep quality be effective. Other reasons that can be considered as influencing children’s sleep in the present study include the decrease in the number of samples, the duration of the intervention, the tensions for fathers during the child’s hospitalization, and the variables affecting fathers’ stress. Although many variables affect the sleep of hospitalized children such as sleeping in a new environment, loud noises, warnings and frequent visits of nurses according to the hospital routine and stress or anxiety of hospitalization^11^, but with the participation of fathers in the form of family-centered care can help the improvement of sleep in hospitalized children.

### Limitation

The noncooperation of families to participate in the study due to the COVID-19 pandemic and the fear of the dangers of participating in the study led to their nonparticipation in the study or their exclusion from the study, which was previously considered a 10% chance of loss. Individual differences of toddlers, environmental disturbing factors such as noise, light, oxygen therapy, etc., were as uncontrollable factors for sleep that were tried to control by random allocation of samples.

## CONCLUSION

COVID-19 pandemic and restricting the presence of families in the hospital has caused the disintegration of families with children hospitalized and the psychological and physical effects of this disintegration are evident in the family members. The results of the study showed that education and support of fathers through social networks and their participation and online training is effective in improving the sleep of hospitalized children with pneumonia. The use of a supportive educational program and helping to restore the parental role of fathers in this study led to improved sleep in hospitalized children with pneumonia, which is an important factor in the recovery process of hospitalized children.
